# Managing acute ischaemic stroke in a small island developing state: meeting the guidelines in Barbados

**DOI:** 10.1186/s12889-018-5565-8

**Published:** 2018-05-22

**Authors:** Asanchia S. Harewood-Marshall, Leslie S. Craig, Tanya P. Martelly, David O. C. Corbin, Lauren Maul, Damani McIntosh-Clarke, Tracey Blackman, Kenneth S. George, Anselm J. M. Hennis, Ian R. Hambleton, Angela M. C. Rose

**Affiliations:** 1 George Alleyne Chronic Disease Research Centre, Caribbean Institute for Health Research, The University of the West Indies, Cave Hill Campus, Barbados; 2 Ministry of Health, Frank Walcott Building, Culloden Road, Bridgetown, Barbados

**Keywords:** Acute stroke, Guideline, Registry, Surveillance

## Abstract

**Background:**

We describe hospital-based management of acute ischaemic stroke patients in 2010–2013 in Barbados, by comparing documented treatment given in the single tertiary public hospital with international guideline recommendations.

**Methods:**

Evidence-based stroke management guidelines were identified through a systematic literature search. Comparisons were made between these guidelines and documented diagnostic practice (all strokes) and prescribed medication (ischaemic stroke only), using a combination of key informant interviews and national stroke registry data for 2010–2013.

**Results:**

Several published international guidelines for the acute management of ischaemic stroke recommended patient management in a dedicated stroke unit or nearest hospital specialised in stroke care. Further, patients should receive clinical diagnosis, CT brain scan, specialist evaluation by a multidisciplinary team and, if eligible, thrombolysis with alteplase within 3–3.5 h of symptom onset. Subsequent secondary prophylaxis, with a platelet aggregation inhibitor and a statin was advised.

Barbados had no stroke unit or stroke team, and no official protocol for acute stroke management during the study period. Most of the 1735 stroke patients were managed by emergency physicians at presentation; if admitted, they were managed on general medical wards. Most had a CT scan (1646; 94.9%). Of 1406 registered ischaemic stroke patients, only 6 (0.4%) had been thrombolysed, 521 (37.1%) received aspirin within 24 h of admission and 670 (47.7%) were prescribed aspirin on discharge.

**Conclusions:**

Acute ischaemic stroke diagnosis was consistent with international recommendations, although this was less evident for treatment. While acknowledging the difficulty in implementing international guidelines in a low-resource setting, there is scope for improvement in acute ischaemic stroke management and/or its documentation in Barbados. A stroke unit was established in August 2013 and written clinical protocols for acute stroke care were in development at the time of the study; future registry data will evaluate their impact. Our findings have implications for other low-resource settings with high stroke burden.

## Background

Chronic non-communicable diseases (NCDs) are the leading cause of morbidity and mortality worldwide [1] and in many developing states, particularly in the Caribbean. A large proportion of this mortality is attributable to cardiovascular disease (CVD), including stroke [2–4].

In 2008, 82% of deaths in Barbados (a small-island nation in the Caribbean with a population of about 278,000 in 2010 [5]) were due to NCDs [6]. Of these, 31% were attributable to CVD, with stroke second only to ischaemic heart disease [6]. Barbados, the fifteenth most densely populated nation in the world [7], is 21 miles long by 14 miles wide, with its single tertiary public hospital situated in the southwest of the island.

In 2008, The University of the West Indies, in collaboration with the Barbados Ministry of Health, implemented a population-based, multi-NCD registry, collecting data on all cases of stroke, heart attack and cancer island-wide [8]. Data for 2009–2013 from the stroke component of this Barbados National Registry for Chronic Non-communicable Disease (the BNR-Stroke) show an incidence rate age-standardised to the World Health Organization (WHO) world 2000 population for all strokes on the island (i.e. not just first-ever events) to be 153 per 100,000 (95% CI 141–166), with age-standardised mortality of 79.3 (95% CI 70.7–88.7) [9] for the same period.

For survivors of stroke, neurological deficits can persist, resulting in profound morbidity and dependence [1, 9] as well as considerable health care costs [10–12] and loss of productivity, which impact at both individual and country levels [10, 11]. Prompt and efficacious management of acute stroke is an important determinant of patient outcome [12] and the efficient allocation of medical resources is critical to ameliorating the impact of stroke on the limited resources of developing countries.

One way of ensuring acceptable levels of care is through the use of standard, evidence-based, clinical practice guidelines (CPGs). Several international CPGs for the management of acute ischaemic stroke have been published within the past decade, primarily by professional service organisations in developed countries [13–23]. However, the degree to which physicians in Barbados (and indeed, in the entire English-speaking Caribbean) adhere to these evidence-based recommendations is unknown. If implemented and managed appropriately, national disease surveillance systems, like the BNR, are an ideal tool for monitoring quality of acute and/or follow-up care. There are very few ongoing stroke surveillance systems in the Caribbean region with published data, particularly for small-island nation states, and none that we could find providing data on treatment. Here we report on the monitoring of acute hospital care for ischaemic stroke in 2010–2013 in Barbados. This work provides the impetus for the attainment of higher standards of stroke care and, by the provision of these baseline data, will allow monitoring of future public health interventions. Also, as the only Caribbean country continuously monitoring national stroke incidence, conducting the first such study of stroke management, we provide important information for other countries in the region.

## Methods

### Aim, design and setting

This study aimed to compare documented stroke management practices recorded by the national stroke registry with time-appropriate international stroke management guidelines, in order to identify areas for improvement, where relevant.

### Evidence for guidelines and current practice

Online databases (UpToDate, Medline, Pubmed and Trip) and four key websites for Caribbean, UK, American and global healthcare guidance were systematically searched for evidence-based recommendations for the management of acute ischaemic stroke (see Appendix), with the final search made on 18 December 2015. Only CPGs published prior to or including the final year of the study (2013) were included, and recommendations from the most current versions applicable during the study period (2010–2013) were identified. This information was summarised into a general consensus from the various CPGs.

Key informant interviews using an informal, semi-structured approach were conducted with acute care physicians to establish current clinical practice for acute stroke in Barbados.

### Data source

Data on stroke management practice were obtained from patients registered with the BNR-Stroke who had been treated at the single tertiary public hospital in Barbados, the Queen Elizabeth Hospital (QEH). The following anonymised data items on acute stroke events treated at the QEH and the management of patients in the acute setting for 2010–2013 (four of the first five full years of registry data collection^1^), were extracted from the BNR-Stroke database: patient demographic information, past medical history, drug history and medical management including admission status, clinical assessments, primary diagnostic tests and vascular imaging (all strokes), specialist evaluations and tests, and pharmacological management on arrival at hospital and on discharge (ischaemic strokes only). In the absence of a stroke unit, hospital ward information was collected to illustrate what proportion of patients received intensive care vs medical ward care.

### Statistical analyses

The number of ischaemic strokes managed at the QEH between 2010 and 2013, mean age, sex and the proportion of patients having the following specific clinical assessments and tests performed were assessed: Glasgow Coma Scale (GCS) score, neurological testing, swallow tests, ECGs and neuroimaging. In addition, the number and proportion of patients having specialist consultations, as well as those being prescribed recommended medications (acutely and on discharge), were assessed. Results were compared with the evidence-based recommendations available up to 2013. In this descriptive study we chose to minimise the number of formal statistical tests performed; where relevant, a test for difference in proportions was used. Data analyses were completed using Stata version 12 (StataCorp., College Station, TX, USA).

## Results

Key informant interviews revealed the absence of a stroke unit and a local stroke management protocol, or one specific international CPG to which all physicians adhered. There was no one standard used by all hospital staff.

### Data description and patient outcomes

There were 2431 acute stroke events registered with the BNR-Stroke between 1 January 2010 and 31 December 2013. Most were managed at the QEH (2001; 82.3%), while the remainder either died before reaching the healthcare setting (408; 16.8%) or were managed in the community (22; 0.9%). Of the 2001 hospitalised patients, 1735 had data fully abstracted by the BNR team (86.7%), while the remaining 13.3% were excluded from analyses as they lacked complete information.

The patients’ ages ranged from 18 to 104 years (mean 69.7 years; SD 15.1), with slightly more females affected than males (51.7% vs 48.3%). Ward information was documented for 1722 patients (99.3%), most of whom (1649; 95.8%) were managed initially by emergency physicians. Over 75% of these were admitted to general medical wards for continued management, with 109 (6.6%) admitted to intensive care units. The remainder were managed on other wards, died in the emergency department (1.2%) or were discharged home (17.8%). Ischaemic stroke was the predominant sub-type (1406; 81.0%), 268 (15.4%) were haemorrhagic strokes and 61 (3.5%) unclassified. Almost three-quarters were discharged from the hospital alive (1007; 71.6%).

### Systematic review of stroke care guidelines

The six CPGs for the management of patients with acute stroke that were publicly available during 2010–2013 included those for the UK [13–16], the USA (with separate guidelines for the management of ischaemic [17] and haemorrhagic [18] stroke in adults), Canada [19, 20], the European Union [21, 22] and Australia [23]. The CPGs all made similar recommendations for the management of acute ischaemic stroke [13–23] summarised under the following headings: (i) Priority response and acute phase care by emergency medical services (EMS) and direct transport to nearest stroke care facility; (ii) clinical diagnosis and management in acute stroke unit; (iii) primary diagnostic tests and vascular imaging; (iv) specialist evaluations; (v) pharmacological management; (vi) surgical interventions; (vii) rehabilitation. In 2010–2013, the QEH stroke unit was not yet implemented and the BNR did not collect information on EMS care. Therefore only parameters (iii)–(v) could be adequately assessed using BNR-Stroke data (see below). The recommendations from each of these three parameters are summarised in Table 1. (A planned future evaluation of the recently implemented stroke unit will assess all seven parameters.)

**Table 1:** Summary of guideline recommendations – diagnosis, specialist assessments and pharmacological management

Recommendations	Further information
Direct admission to specialised stroke unit	
Diagnostic tests	Neuroimaging (non-contrast CT/MRI)
Secondary diagnostic tests	ECG and other cardiac investigations if indicated Vascular imaging, e.g. CT/MR angiography and duplex ultrasonography
Specialists’ assessments	Neurologist, nutritionist, physiotherapist, speech therapist, rehabilitation therapists
Pharmacological management	
Thrombolysis	Intravenous alteplase within 3–4.5 h of symptoms for fibrinolysis
Antiplatelet therapy	Aspirin (acetyl salicylic acid) primarily (alternates: Clopidogrel or Dipyridamole) Acute use (associated with slight reduction in mortality and morbidity) Prescribed on discharge (secondary prophylaxis)
Anticoagulants	Avoid routine use except if at risk of thromboembolism
Statins	Avoid routine use Advised for secondary prophylaxis

### Primary diagnostic tests and vascular imaging (all strokes; *N* = 1735)

Following clinical diagnosis of stroke, urgent neuroimaging was recommended by all CPGs [13–23] to identify stroke type and guide treatment. Computed tomography (CT) was the primary diagnostic scan recommended (lower cost and shorter duration), but most guidelines noted that magnetic resonance imaging (MRI), if available, was useful for detecting early signs of ischaemia. Additional vascular imaging (carotid ultrasound examination, cerebral/carotid angiography) were also recommended [13–23].

The only form of neuroimaging available at the QEH was the CT brain scan, with unenhanced CT scans performed for most patients (1646; 94.9%); 1117 of them (67.9%) within 24 h of hospital arrival. Any MRIs were done privately (and non-acutely); these were performed for only 18 (1.0%) patients. Vascular imaging was also performed infrequently (Table 2).

**Table 2:** Primary and secondary diagnostic tests at the Queen Elizabeth Hospital, Barbados, 2010–2013 (*N* = 1735)

Recommended investigation		Tests performed	
		Number	%
CT scan		1646	94.9
MRI		18	1.0
CT scan/timing	≤ 24 h	1117	67.9
	> 24 h	496	30.2
Electrocardiography		1527	88.0
Echocardiography		55	3.2
Cerebrovascular angiography		36	2.1
Carotid ultrasound		32	1.8
Carotid artery angiography		14	0.8

### Specialist evaluations (ischaemic strokes; *N* = 1406)

All six CPGs [13–23] advocated multidisciplinary management, requiring patients to be evaluated within the first few hours of admission by a number of specialists (for example neurologist, dietician, physiotherapist) to identify their specific needs for acute care, nutrition and rehabilitation.

For the 1406 ischaemic stroke patients, neurological examinations, including dysphagia screening (776; 55.2%) were performed primarily by physicians who were not neurologists. Less than half of the patients had documentation of neurologist consultations (117; 44.5%), 50.2% (409) were seen by physiotherapists and very few received other specialist assessments (Table 3).

**Table 3:** Specialist consultations for ischaemic stroke patients at the Queen Elizabeth Hospital, Barbados, 2010–2013

Specialist consultation^a^	N^b^	Patients assessed	
		No.	%
Physiotherapist	815	409	50.2
Neurologist evaluation	263	117	44.5
Cardiologist	153	30	19.6
Speech and language pathologist	263	29	11.0
Occupational therapist	170	17	10.0
Rehabilitation specialist	139	13	9.4
Neurosurgeon evaluation	0	0	0

^a^Data on nutritionist consultations were not abstracted by the registry

^b^Total number of patients for whom data were available

### Pharmacological management (ischaemic strokes)

This study focused on acute treatment (given within 24 h of the onset of stroke symptoms or within 24 h of arrival at QEH) and drugs prescribed on discharge.

#### Thrombolysis

**Fig. 1:**
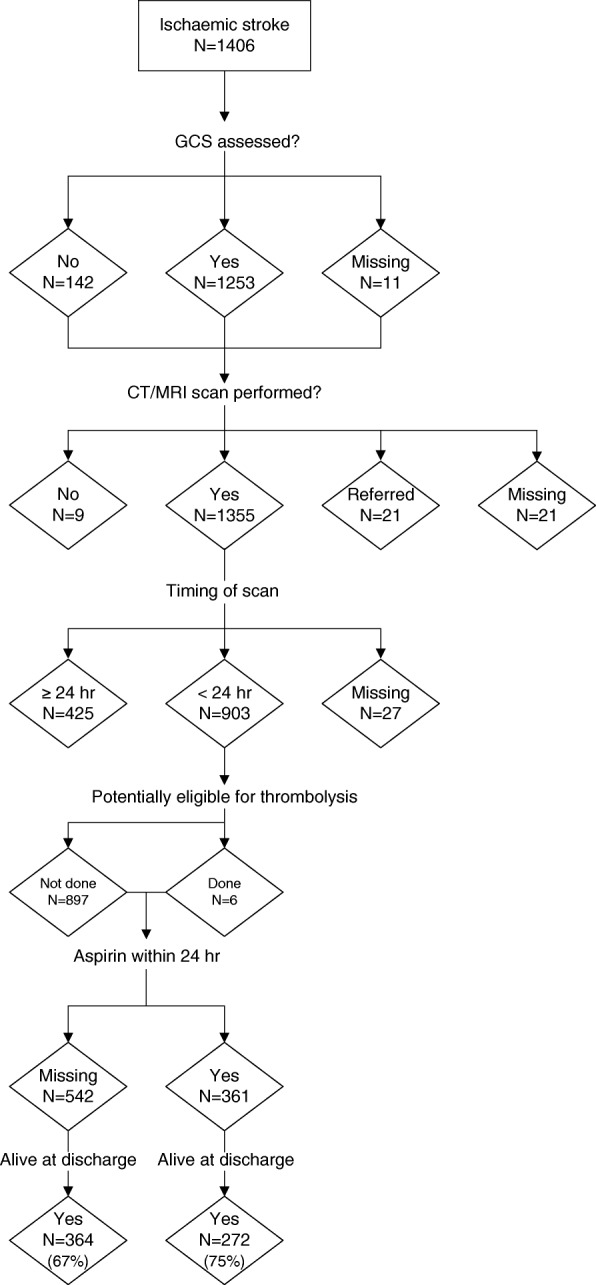
Acute management and outcomes for ischaemic stroke patients in Barbados, 2010–2013

All CPGs [13–23] recommended that eligible patients receive the intravenous thrombolytic agent alteplase within 3–4.5 h of stroke symptom onset. Only six patients received intravenous thrombolysis between 2010 and 2013 (Table 4), out of 903 potentially eligible patients (Fig. 1).

**Table 4:** Drugs prescribed for hospitalised ischaemic stroke patients, Barbados, 2010–2013

Drug category	Drug name	Prescribed acutely		Prescribed on discharge	
		No.	%	No.	%
Antiplatelet drugs	Aspirin	521	37.0	670	47.7
	Clopidogrel	50	3.6	66	4.7
	Dipyridamole	< 10	< 1	13	0.9
	Aggrenox	< 10	< 1	12	0.9
Fibrinolytic	Alteplase	< 10	< 1	n/a	
Anticoagulants	Warfarin	16	1.1	49	3.5
	Heparin (subcutaneous)	333	23.7	n/a	
	Heparin (low molecular weight)	44	3.1	32	2.3
Statins	Simvastatin, Atorvastatin	304	21.6	681	48.4

*n/a* not applicable

#### Antiplatelet therapy

All CPGs recommended aspirin [13–23] or clopidogrel [16] as acute antithrombotic therapy to improve stroke outcome. Continuation of the drug or combinations of the drug with dipyridamole [14] after discharge was advised for secondary prophylaxis against recurrence. Alternative agents recommended for those intolerant of aspirin, or for whom it was contraindicated, included clopidogrel and dipyridamole [21, 22]. In Barbados, less than 50% of hospitalised ischaemic stroke patients were documented as having received aspirin acutely or upon discharge. Other antiplatelet drugs available at the QEH during 2010–2013 were clopidogrel, dipyridamole, and Aggrenox (aspirin plus modified dipyridamole); however, these were infrequently prescribed (< 5%; Table 4).

Of the 903 (64.2%) ischaemic stroke patients who received a CT scan within 24 h, only 40.0% were documented as having been given aspirin within that time (Fig. 1). The case fatality in ischaemic stroke patients who had been documented as having received aspirin acutely (24.7%; 95% CI 20.3–29.1) was statistically significantly lower than those who had not (32.8%; 95% CI 28.9–36.8; *p* = 0.009) (Fig. 1).

#### Anticoagulants

All guidelines [13–23] discouraged the use of anticoagulants except in selected patients which included those at risk of thromboembolic phenomena (e.g. with atrial fibrillation or thrombophilias) [13–23] and patients with cerebral venous sinus thrombosis [14]. In Barbados, 1.1% of ischaemic stroke patients were given warfarin acutely, 3.5% received the drug on discharge (Table 4) and 48 patients were chronic users (data not shown). In each successive year, acute use was reduced; patients continuing warfarin likely had high risk of recurrent stroke (e.g. co-morbid atrial fibrillation: 107 patients). Acute use of heparin was documented for 377 (26.8%) patients (Table 4).

#### Statins

Most guidelines did not recommend the initiation of statins in the acute setting, but supported their continuation for patients with prior chronic use. All guidelines [13–23] recommended the subsequent use of statins as secondary prophylaxis against recurrent ischaemic stroke. Acutely, 304 (21.6%) hospitalised patients in Barbados were documented as having received statins while 681 (48.4%) were prescribed a statin on discharge (Table 4).

## Discussion

Our study showed that, during 2010–2013, the diagnostic approach to acute stroke at the QEH in Barbados was consistent with international CPGs. Stroke was diagnosed clinically, and almost all stroke patients (95%) had documented CT brain imaging during their hospital stay; 68% within 24 h. Unfortunately, the exact timing of CT scans was not collected by the registry (which just has a checkbox for “CT within 24 hrs”), thus we were unable to determine the proportion of patients scanned within 3–4.5 h of symptom onset. Our study showed that, in contrast to what was found for diagnostics, the management of patients with ischaemic stroke was not in keeping with CPG recommendations, as these patients were managed mainly on general medical wards during the study period. During the study period, few persons were documented as having received the specialist consultations recommended by the CPGs as part of routine ischaemic stroke care. Further, thrombolysis was seldom performed, with fewer than 10 patients thrombolysed in 2010–2013. In Barbados, few patient records (37%) documented the acute use of aspirin, despite evidence in support of aspirin therapy causing minor improvements in dependence and mortality when given within 24 h of the event. Fewer records than expected (< 50%) showed that aspirin had been given as secondary prophylaxis on discharge. Likewise, there was limited documentation of prescription of statins for secondary prophylaxis of ischaemic stroke. Despite this, our study found that the overall proportion of hospitalised ischaemic stroke patients who survived to 30 days was 74.9%, for a mean 30-day case fatality of 24.9% (22.7–27.3).

Our rate of administration of CT scans within 24 h is similar to the 69% seen in Canada [24] and is commendable given the absence of a specific, written hospital stroke protocol. However, there is still considerable room for improvement, especially when compared with other countries such as Australia, where 89% of patients receive CT scans within 24 h [25].

In Barbados, late patient presentation to the hospital, the lack of a protocol for administering alteplase for stroke, and the absence of a stroke unit in which to subsequently manage patients, may have collectively accounted for the low rates of thrombolysis, which have also been noted by other low-resourced countries [26, 27]. Even hospitals equipped with better developed stroke care systems experience challenges, however. The 2011 Australian National stroke audit found only 36% of patients presented to facilities within 3 h of stroke symptom onset, only 7% of patients were thrombolysed and less than 60% of patients were managed in stroke units [28]. Similarly, in Canada, only 25% of hospitals providing acute stroke care had a specialised programme; 17% had stroke units and 50% of patients presented to the hospital outside the thrombolysis eligibility period [24]. Conversely, other countries (e.g. England, Wales and Northern Ireland) found marked improvements in CT scan times (43% in 1 h, 80% in 12 h) and thrombolysis rates (75%) [29]. Award programmes such as the AHA/ASA 2013 ‘Get with the guidelines – Stroke’^(R)^, have proved useful in driving improvement in some American hospitals. Almost 60% of hospitals participating in this initiative achieved the door-to-needle target for thrombolysis of 60 min or less [30].

Interviews with the sole neurologist at the hospital in Barbados suggested that our observations on low proportions of aspirin and other key medications are unlikely to be a true reflection of clinical practice, more likely being due to omissions in documentation. Improved documentation in medical records is clearly needed, not only for surveillance, medico-legal and research purposes, but also to establish a true reflection of patient management and ensure best practices are being consistently followed. Care by a specialised stroke team and, where possible, care in a dedicated stroke unit (both recommended as first-line treatment by all CPGs) are associated with reduced mortality and patient disability [29, 31, 32].

Low levels of specialist input and rehabilitation therapy likely reflect a lack of CPG guidance or poor documentation, as well as limited resources and/or ineffective resource allocation. For example, there was only one neurologist and a limited number of therapists working at the hospital, presenting an impractical patient load. Only 117 patients had documented evidence of referral to the neurologist and a mere 37 to a speech and language therapist (Table 3). Although improved patient outcomes are associated with adherence to CPGs [25, 33], the goal of such guidelines is not strict adherence but rather to provide physicians with minimum basic standards and proven treatment algorithms that can be tailored to the individual needs of specific patients. However, CPGs for developed countries may not always be appropriate or implementable in low-resourced, small-island developing states such as Barbados. For example, general physicians managing stroke patients may only refer difficult cases for specialist consultation and may not recognise subtle deficits that may be critical to determining patient management. The lack of expert assessment of dysphagia by neurologists could lead to high levels of aspiration pneumonia and higher mortality.

Data from a 2006 study comparing acute stroke outcomes for Black Barbadians and a similar population in South London revealed relatively poor first-ever stroke survival for the island (hazard ratio 1.99; 95% CI 1.23–3.21, *p* = 0.005) [34]. Comparisons of local acute stroke management and post-stroke care subsequent to discharge were beyond the scope of this study, which only analysed data collected by the national stroke registry. The case fatality we found, however, is comparable with the estimated worldwide 30-day case fatality after first ischaemic stroke (16–23%) [33] and with rates for other Caribbean countries, such as Trinidad (29%) [27], suggesting that ischaemic stroke patients in Barbados received at least a similar standard of care to patients in countries with similar resources. However, improvement is needed given the far lower case fatalities observed in developed countries during the same period (e.g. United Kingdom: 12.9% [29], Canada, excluding Québec: 15% [29], USA: 4.2% [26]).

The limitations of our study included the retrospective (secondary) use of prospectively collected data acquired solely from hospital records, which firstly limited our analyses to only those parameters already collected by the registry. Secondly, our analysis would have failed to capture any undocumented consultations and treatments. Poor documentation of key parameters, e.g. aspirin use, may have skewed the results obtained, and it is impossible to determine whether care was actually omitted, or given but not recorded. Neither option reflects good clinical practice. The BNR team will continue their efforts to address the issue of poor documentation during professional development seminars for medical professionals. Future years of BNR data will be able to assess the impact of the stroke unit on stroke outcomes in Barbados.

## Conclusion

Despite good adherence to diagnostic CPGs, there was a lack of evidence of adherence to standard CPGs for acute medical management of ischaemic stroke between 2010 and 2013 in Barbados, which may be due in part to the limited resources experienced by small-island developing nations. Mitigating the mortality and degree of morbidity experienced by patients is dependent on rapid appropriate management from the time of symptom onset. It is therefore essential that patients be aware of the symptoms of stroke, as well as the importance of reporting immediately to a medical facility for evaluation and subsequent management. This speaks to the need to educate the general public through health promotion information on stroke, as well as the direct education of at-risk patients by primary care providers.

The recent implementation of a stroke unit at the hospital, as well as future hospital stroke management protocols, will be important steps toward improving the standard of care at the QEH. Monitoring of these initiatives will be provided through continued stroke surveillance. Results from this study likely reflect practices in other similar, low-resource settings, with implications particularly for other Caribbean nations.

## Appendix

### Details of literature search

The following databases were searched online for published literature:

UpToDate.

PUBMED/MEDLINE.

Trip.

For ‘grey’ literature (i.e. technical and/or government reports which may not be published in the scientific literature), the following websites were reviewed:

Centers for Disease Control and Prevention (CDC), USA. http://www.cdc.gov/

World Health Organization (WHO). http://www.who.int/en/

Caribbean Health Research Council (CHRC). http://carpha.org

National Institute of Health for Clinical Excellence (NICE). http://www.nice.org.uk/

The following terms were used for each database/website search:

acute stroke management

ischaemic haemorrhagic stroke management

stroke best practice

stroke guidelines

stroke management guidelines

stroke treatment

stroke treatment recommendations

Databases and websites were last searched on 18 December 2015.

## Abbreviations

AHA: American Heart Association
ASA: American Stroke Association
BNR: Barbados National Registry for Chronic Non-communicable Disease
CDC: Centers for Disease Control and Prevention
CHRC: Caribbean Health Research Council
CPG: Clinical practice guideline
CSN: Canadian Stroke Network
CT: Computed tomography
CVD: Cardiovascular disease
ECG: Electrocardiogram
EMS: Emergency medical services
ESO: European Stroke Organisation
GCS: Glasgow coma scale
HSF: Heart and Stroke Foundation
ISWG: Intercollegiate Stroke Working Group
MRI: Magnetic resonance imaging
NCD: Non-communicable disease
NICE: National Institute for Health and Care Excellence
QEH: Queen Elizabeth Hospital
SD: Standard deviation
SIGN: Scottish Intercollegiate Guideline Network
UK: United Kingdom
USA: United States of America
WHO: World Health Organization

## Footnotes

1 Note: data from 2009 were excluded, as it was the first registry year.
